# Advances in Through-Hole Anodic Aluminum Oxide (AAO) Membrane and Its Applications: A Review

**DOI:** 10.3390/nano15211665

**Published:** 2025-11-01

**Authors:** Chin-An Ku, Chen-Kuei Chung

**Affiliations:** Department of Mechanical Engineering, National Cheng Kung University, Tainan 701, Taiwan

**Keywords:** anodic aluminum oxide, AAO, membrane detachment, SERS, TENG

## Abstract

Anodic aluminum oxide (AAO) is a well-known nanomaterial template formed under specific electrochemical conditions. By adjusting voltage, temperature, electrolyte type, and concentration, various microstructural modifications of AAO can be achieved within its hexagonally arranged pore array. To enable broader applications or enhance performance, post-treatment is often employed to further modify its nanostructure after anodization. Among these post-treatment techniques, AAO membrane detachment methods have been widely studied and can be categorized into traditional etching methods, voltage reduction methods, reverse bias voltage detachment methods, pulse voltage detachment methods, and further anodization techniques. Among various delamination processes, the mechanism is highly related to the selectivity of wet etching, as well as the Joule heating and stress generated during the process. Each of these detachment methods has its own advantages and drawbacks, including processing time, complexity, film integrity, and the toxicity of the solutions used. Consequently, researchers have devoted significant effort to optimizing and improving these techniques. Furthermore, through-hole AAO membranes have been applied in various fields, such as humidity sensors, nanomaterial synthesis, filtration, surface-enhanced Raman scattering (SERS), and tribo-electrical nano-generators (TENG). In particular, the rough and porous structures formed at the bottom of AAO films significantly enhance sensor performance. Depending on specific application requirements, selecting or refining the appropriate processing method is crucial to achieving optimal results. As a versatile nanomaterial template, AAO itself is expected to play a key role in future advancements in environmental safety, bio-applications, energy technologies, and food safety.

## 1. Introduction to Nanoporous Membrane for Science and Engineering

**Figure 1:**
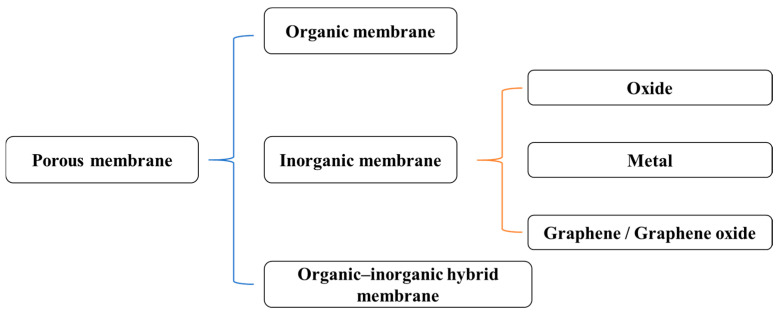
Simple classification of nanoporous membranes.

Nanoporous membranes are an important class of materials with tunable porosity, high surface area, and diverse chemical functionality [1]. These membranes serve critical roles in applications such as optical and electrical sensing, catalysis, nanomaterial synthesis, filtration, and separation, as well as energy applications [2,3,4,5]. With increasing demands, nanoporous membranes have been extensively employed across a wide range of fields, including scientific research, industry, food and environmental safety, nanotechnology, water purification, textile manufacturing, and our daily life [1,2,3,4,5,6]. These membranes can be broadly categorized into organic, inorganic, and organic–inorganic hybrid materials [1], as shown in Figure 1.

In terms of organic materials, including polymers such as polyethersulfone, polyvinylidene fluoride, polyacrylonitrile, polyamide, and Polytetrafluoroethylene (PTFE), they offer high flexibility in design and functionalization. Nanoporous polymer membranes are often processed through nano-imprinting, electrospinning, interfacial polymerization, or chemical synthesis [7,8,9]. These films are especially suitable for water purification, biocompatible membranes, and flexible electronics, with the added benefit of tunable chemical affinity and porosity.

Oxide membranes are typically composed of materials such as porous silica, AAO, or TiO_2_ [10,11,12,13,14]. They exhibit excellent thermal and mechanical stability, high surface area, and well-defined pore structures. Fabrication methods include sol–gel processes, template-assisted deposition, and anodization. However, the morphology of porous silica membrane tends to resemble nanoparticles or nanospheres, which is still distinct from the vertically aligned pore structures of AAO and TiO_2_. Porous silica is primarily used in gas adsorption, separation, and biomedical or sensing applications [15,16,17]. Porous metal templates, like their oxide counterparts, are classified as inorganic templates [18,19,20,21]. The primary fabrication methods include deposition onto oxide templates and dealloying. However, precise control of pore size in metallic systems can be challenging. Graphene oxide is another commonly used inorganic porous membrane, characterized by its high specific surface area, excellent hydrophilicity and biocompatibility, and tunable mechanical and electrical properties [22,23,24,25,26]. It is typically fabricated through hydrothermal synthesis, sol–gel processes, or chemical vapor deposition (CVD). Porous graphene films are suitable for electrical and optical sensing, substance separation, and filtration, particularly in the field of water purification [22,23,24,25,26]. Carbon nanotubes (CNTs) are another promising material with controllable tube diameters. Their fabrication methods mainly include powder-based chemical synthesis, laser sintering, and CVD. Notable applications include water purification, energy storage, gas separation, and sensing [27,28,29].

Inorganic–organic hybrid membranes have become a growing trend in research. By incorporating various materials, the physical and chemical properties as well as the thermal stability of the membranes can be effectively modified, enabling broader applications and enhanced performance. Both top–down and bottom–up approaches can be used to fabricate inorganic–organic hybrid membranes, including methods such as grinding, laser sintering, sputtering, etching, chemical deposition, CVD, sol–gel processing, and electrospinning [1,30,31,32,33]. These materials hold great promise in gas sensing, separation membranes, and energy application [34,35,36,37].

Among various types of nanoporous membranes, structural control of metal and polymer membranes remains relatively challenging. GO membranes, CNT, and inorganic–organic hybrid nanoporous membranes, on the other hand, typically require long fabrication time and complex processes, which raise the barrier for practical applications. In contrast, AAO membranes offer several advantages, including high thermal stability, biocompatibility, self-organization, vertical growth with high aspect ratio structure, and tunable, highly ordered nanostructures. Compared with other inorganic oxide membranes such as porous silica and titanium dioxide, AAO also benefits from relatively lower production costs, contributing to its widespread use. Furthermore, AAO templates can be used for nanomaterial synthesis or nanopattern transfer to produce metallic or polymeric nanomaterial films. These advantages make AAO membranes highly promising for various applications, and the development of methods for detaching AAO membranes to enable broader utilization has drawn significant attention from many research groups.

### 1.1. Anodic Aluminum Oxide (AAO) Through-Hole Membrane

**Figure 2:**
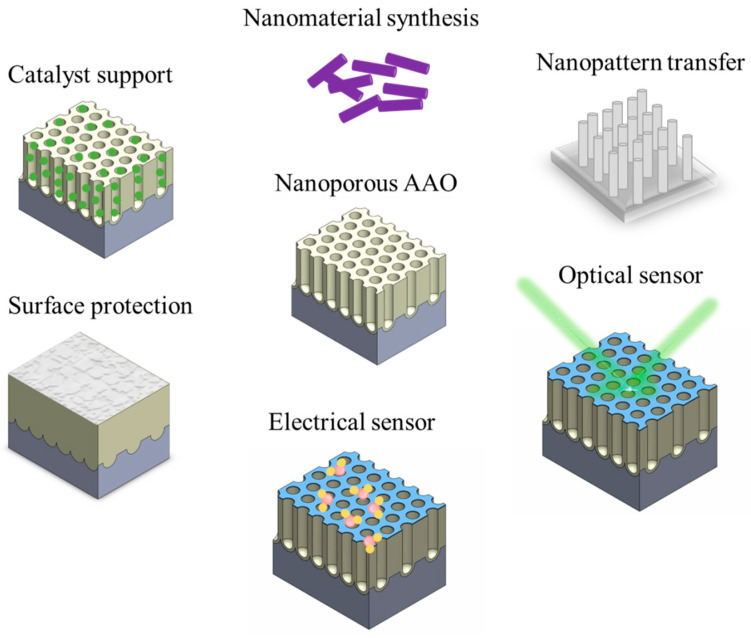
Common applications of porous AAO membranes on bulk Al substrate.

Anodic aluminum oxide (AAO) is a nanomaterial template formed under specific electrochemical conditions. Due to its high aspect ratio structure, tunable nanostructures, ease of fabrication, relatively low cost, good thermal stability, and biocompatibility, it has been widely used in both research and industry [38,39,40,41,42]. AAO is a well-developed technology with extensive applications, as shown in Figure 2. In industrial applications, AAO is primarily used for surface protection in 3C products and aircraft [43,44,45,46,47,48], with research focusing on enhancing hardness [49,50,51,52,53], corrosion resistance [54,55,56,57,58], and surface properties [59,60,61,62,63,64,65,66]. In research, the applications of AAO are even broader, covering nanomaterial synthesis [10,67,68,69], nanopattern transfer [70,71,72,73,74], electrical sensors [75,76,77,78,79,80], surface-enhanced Raman scattering [81,82,83,84,85,86], optical devices [87,88,89,90,91], photocatalysis [92,93,94,95,96], bio-applications [97,98,99,100,101], and other fields [102,103,104,105,106,107,108]. To achieve effective applications across different domains, adjusting the fabrication parameters of AAO to obtain diverse nanostructures is a key research direction [38,39,40,109,110,111].

**Figure 3:**
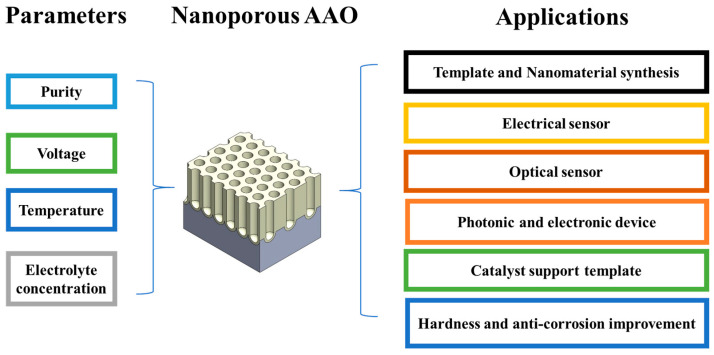
Overview of porous AAO parameters and applications. (From Ku et al., used under CC-BY 4.0 license [38]).

The overview of anodization parameters and applications is provided in Figure 3. The main tunable parameters for AAO include the applied voltage, type and concentration of acid, temperature, anodization time, and type of aluminum substrate [38,39,40,41,42]. The applied voltage significantly influences the pore diameter (D_p_), interpore distance (D_int_), and film thickness. Specifically, the applied voltage is directly proportional to the interpore distance and also positively correlates with the pore diameter. Based on the effect of voltage on anodization, the process can be categorized into mild anodization and hard anodization. Mild anodization typically refers to the conventional low-temperature DC anodization process. However, due to the varying dissociation degrees of different electrolytes, the anodization voltage also differs. Commonly used voltages for various electrolytes including sulfuric acid at 20–25 V [112], oxalic acid at 30–40 V [113], and phosphoric acid at 160–195 V [114]. On the other hand, hard anodization proposed by Lee et al. [115] involves applying a higher voltage than mild anodization, which enables a higher growth rate and faster AAO fabrication [116,117,118]. For hard anodization, sulfuric acid is typically used at 40–70 V and oxalic acid at 100–150 V [115].

In terms of temperature and time, traditional low-temperature (0–10 °C) anodization is more favorable for forming complete and well-ordered AAO pores. This is attributed to low temperature, which reduces the chemical dissolution rate at the interface between oxide layer and electrolyte. Additionally, thermal exchange is effectively dissipated by lower temperatures and prevents the burning effect from Joule heating generated at the pore bottom. However, low-temperature processes also suppress the AAO growth rate, resulting in lower current density, which directly affects the film thickness and growth rate. As a result, many researchers aim to raise the anodization temperature to room temperature to shorten the process time [38,83,84,119].

Moreover, different aluminum substrates contain various elements, which affect the nanostructure during anodization of different alloys. For example, Moon et al. [50] reported that AAO formed on 1xxx and 5xxx series aluminum alloys grows significantly faster than on 2xxx and 6xxx series alloys due to differences in alloy elements.

Although many studies have investigated the influence of parameters like pore diameter and interpore distance on AAO structure, certain nanostructures still cannot be achieved solely through the adjustment of these anodization parameters. For example, achieving structures with larger pore diameters but the same interpore distance with higher specific surface area often requires the assistance of a pore-widening process [120]. Additionally, to enhance surface protection properties such as high hardness and corrosion resistance, many research groups have implemented pore-sealing treatments [51,52,53] to improve performance. To obtain more diverse nanostructures, various post-treatment methods have attracted significant attention. In addition to the mentioned pore-widening and sealing processes, AAO membrane detachment has also become a topic of great interest. The through-hole AAO membrane possesses unique 2D–3D structures not found on the top surface. After removing the aluminum substrate, applications such as mask or filtration not achievable with complete AAO membranes are proposed. Traditionally, AAO membrane detachment has mostly relied on heavy metal solutions containing Hg^2^^+^ or Cu^2^^+^ to remove the aluminum substrate and obtain the free-standing AAO [113,121,122,123,124,125,126,127,128,129,130,131,132,133]. However, to prevent environmental pollution and reduce material waste, current studies are actively developing fast and eco-friendly alternatives. In this article, we will begin with a review of related developments, followed by a detailed discussion of AAO membrane detachment methods and related applications in Section 2 and Section 3.

### 1.2. Brief Development of AAO

AAO is a well-known nanomaterial template, and its development has long been intertwined with the rise in nanotechnology. There are several key milestones in the evolution of AAO and its membrane detachment methods. In 1953, Keller et al. [134] proposed the hexagonal structure model of anodic aluminum oxide, taking a pioneering role in the study of AAO nanostructures. In 1963, Spooner [135] was the first to propose a voltage-based separation method using perchloric acid solution to detach AAO membranes, marking the earliest non-etching approach documented in the literature. In 1989, Rigby et al. [136] introduced the voltage step-down method, where the barrier layer of AAO was gradually thinned and disrupted by stepwise voltage reduction, promoting the detachment of the AAO membrane from the aluminum substrate. In 2005, Schneider et al. [137] proposed the reverse bias method for AAO membrane separation, which enabled repeated recovery of intact membranes. By applying a negative voltage, hydrogen gas was generated at the AAO–aluminum interface, facilitating separation. The resulting membrane retained the barrier layer, and predefined traces remained on the aluminum substrate. In 2006, Lee et al. [115] introduced the hard anodization method, which allowed for faster AAO membrane fabrication. In 2009, Qiu et al. [138] conducted research using AAO as a SERS substrate. However, since the front side of the AAO membrane was used, the enhancement factor (EF) was relatively low, at only about 10^4^. In 2015, Masuda et al. [139] proposed the further anodization method, in which a sacrificial layer was grown to achieve intact membrane separation while preserving the aluminum substrate for repeated use. In 2019, Sulka et al. [140] utilized a pulsed voltage method in perchloric acid solution to achieve membrane separation. They were the first to successfully obtain a through-hole AAO membrane under hard anodization conditions in sulfuric acid and presented a mostly intact membrane image.

### 1.3. Post-Treatments After Anodization

**Figure 4:**
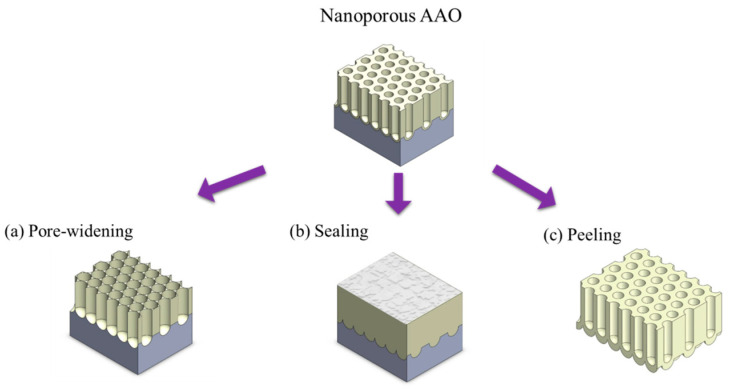
Common post-treatment methods for porous AAO, including (**a**) pore widening, (**b**) pore sealing, and (**c**) membrane peeling.

In anodization, certain parameters interact with each other; for instance, voltage and temperature both influence the membrane thickness. Some parameters affect multiple geometrical features of the pores, for instance, voltage impacts interpore distance, thickness, and pore diameter. These complex interdependencies make it challenging to achieve specific structures. Therefore, post-treatment after the anodization process is of significant research importance. The most common AAO post-treatments can be categorized into three types, including pore widening, pore sealing, and membrane detachment, as shown in Figure 4.

For example, creating high-surface-area structure is positively correlated to large pore diameter. However, raising anodization voltage also increases the interpore distance, which negatively affects the specific surface area. Therefore, a pore-widening process is needed as assistance. Pore widening [141,142] can increase both the pore diameter and specific surface area [120], which is beneficial for applications such as nanomaterial synthesis, nanopattern transfer, and the enhancement of electrical and optical sensor performance [10,143,144].

During AAO growth, the reaction occurs between the barrier layer and the aluminum substrate, inevitably resulting in pore formation. By achieving a balance between dissolution and deposition, the pores enable continuous downward growth of the AAO, thereby increasing its thickness [38]. However, for surface protection applications, such as achieving high surface hardness and corrosion resistance, reducing porosity is essential. Since hardness is contributed by the AAO itself, minimizing the air proportion within the AAO layer enhances surface hardness [49,50,51,52,53]. On the other hand, in corrosion testing, the barrier layer at the bottom of the AAO pores is the location with lowest electrical resistance. Therefore, reducing porosity also significantly improves corrosion resistance. To fully fill the AAO pores and achieve surface protection, a pore-sealing process is necessary, and is generally achieved by boiling water, oil, or salt-compound sealing [52,53,145,146,147,148,149].

Another difficult challenge in AAO research is the fabrication of 3D nanostructures [84,86,150]. Unlike the planar, well-ordered AAO structures, 3D AAO structures are irregular and more difficult to fabricate. Current approaches include surface roughening of the aluminum substrate, using AAO as a template to synthesize other materials, or employing post-treatment methods such as membrane detachment. Although most AAO applications rely on the ordered nanostructures of AAO, certain applications, such as SERS, prefer irregular 3D structures with more hotspots. In such cases, AAO membrane detachment becomes a promising direction for research. The bottom of through-hole AAO membranes features a regular 2D–3D nanostructure, and multiple studies have confirmed its contribution to enhancing SERS signals [151,152,153].

### 1.4. Applications Based on AAO Through-Hole Membrane

The applications of through-hole AAO membranes include electrical sensors [154,155], nanomaterial synthesis [155,156,157], photomasks [158], filtration [159,160,161,162,163], SERS [151,152,153], and triboelectric nano-generators (TENG) [164,165,166], which differ slightly from AAO on substrates. Through-hole AAO membranes are hard and brittle, making them unsuitable for surface protection applications. Their thickness typically ranges from 10 to 100 μm, far exceeding the visible light wavelength of 400–700 nm, which limits their use in structural color optical components and reflectometric interference sensors (RIfS). On the other hand, the through-hole and light-transmitting properties of the membrane enhance its potential in filtration and photomask applications. Additionally, the 2D–3D nanostructures at the bottom of the through-hole membranes significantly increase the number of hotspots, further improving the performance of SERS detection. In AAO-TENG research, AAO templates are commonly used for nanopattern transfer to fabricate TENGs [164,165]. The tunable nanopore structure and density of AAO can effectively enhance the performance of TENG. However, some research teams have also proposed grinding AAO membrane into powder for doping [166], which can effectively enhance TENG performance. The detailed methods for AAO membrane detachment and their applications will be thoroughly discussed in Section 2 and Section 3.

## 2. AAO Membrane Detachment Methods

AAO membranes feature a well-ordered nanoporous array, making it suitable in applications of nanomaterial synthesis, filtration, photomasks, substrates for SERS, and integration with TENGs. As a result, many researchers have focused on the detachment of AAO films. Traditionally, AAO membrane detachment has been achieved using heavy metal solutions containing mercury or copper ions to etch away the aluminum substrate. However, to prevent environmental pollution and material waste, research teams have increasingly avoided this approach and have instead developed alternative methods such as the voltage reduction method [136], reverse bias voltage method [137,156,157,167], pulse voltage method [135,140,168,169,170], two-layer anodization method [139,158,171,172], and constant voltage detachment method [173,174,175]. This chapter will explore each of these techniques in detail.

### 2.1. Traditional Etching Method

Traditional etching method to remove the aluminum substrates using a solution containing Hg^2^^+^ or Cu^2^^+^ to obtain AAO membrane is the most common method for AAO membrane detachment [113,121,122,123,124,125,126,127,128,129,130,131,132,133,176,177]. A summary of related methods is provided in Table 1. The traditional etching method has been widely applied and remains the standard process for commercial AAO film fabrication, primarily because it enables the retrieval of intact, undamaged AAO membranes. It is highly important for applications such as nanomaterial synthesis, filtration, and SERS.

However, this approach requires the use of heavy metal solutions, resulting in highly toxic waste and environmental concerns. To avoid the use of toxic heavy metal solutions, some studies have adopted saturated iodine methanol solution for membrane separation [178,179]. HgCl_2_, CuCl_2_, and I_2_-based solutions exhibit high selectivity toward aluminum without etching alumina, and thus have been widely used in many studies. The main advantage of this method is that it introduces little mechanical stress during the process, allowing for the retrieval of large-area and complete AAO membranes. However, aside from environmental concerns, this method leaves behind the AAO barrier layer, necessitating extra steps to achieve the desired through-hole membrane. It also induces pore-widening effects on the AAO, and whether this affects the nanostructure and its applications significantly may vary between studies. In addition, this method requires complete etching of the aluminum substrate, leading to substantial material waste. The process also takes longer and varies depending on the thickness of the aluminum substrate. This makes the process complex and time-consuming, prompting many researchers to actively develop improved separation techniques.

### 2.2. Voltage Reduction Method

Voltage reduction detachment technique was proposed by Rigby et al. in Nature in 1989 [136]. The AAO barrier layer was gradually broken down by stepwise decreasing the applied potential from 160 to 0.1 V, ultimately facilitating the detachment of AAO membrane from aluminum substrate. In voltage-based separation methods, the stepwise voltage reduction method is characterized by its ability to detach AAO formed at higher voltages. By thinning the AAO barrier layer, this approach enables pore opening without causing pore widening. Meanwhile, thinner AAO barrier layer exhibits lower electrical resistance, and some studies have utilized this approach for electrodeposition in nanomaterial synthesis applications [180]. However, this method is relatively time-consuming, and the voltage adjustments lack a standardized guideline, relying mostly on experimental experience. Therefore, relatively few research groups have adopted this method.

### 2.3. Reverse Bias Voltage Detachment Method

**Figure 5:**
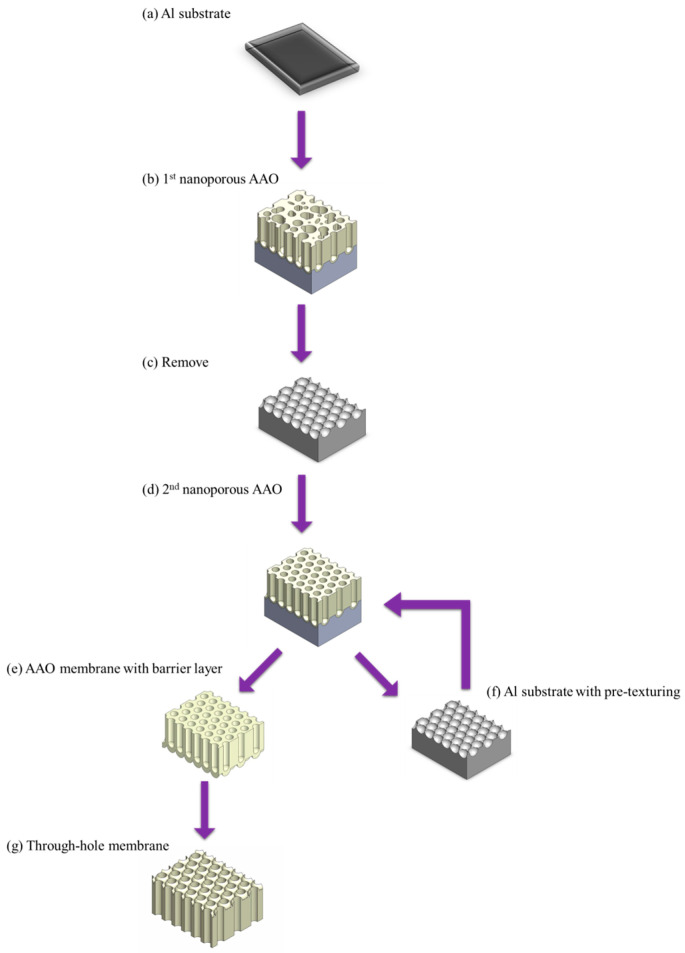
The process flow of AAO membrane prepared by reverse bias voltage detachment method, including (**a**) Al substrate, (**b**) 1st anodization with disordered nanoporous AAO, (**c**) remove and leave pre-texturing Al surface, (**d**) 2nd anodization with ordered nanoporous AAO, (**e**) free-standing AAO membrane after detachment process, (**f**) substrate after AAO detachment, and (**g**) through-hole AAO membrane.

The reverse bias voltage method achieves AAO membrane detachment by applying a small negative voltage, generating hydrogen gas bubbles between the aluminum substrate and AAO barrier layer. These bubbles push AAO membrane away, resulting in a complete membrane and Al substrate with pre-texturing. In 2005, Schneider et al. [137] successfully demonstrated AAO membrane detachment using this method, and proposed the standard process flow. AAO fabricated in sulfuric acid at 25 V and in oxalic acid at 40 and 50 V resulted in pore diameters of 33, 40, and 72 nm, respectively. In the same year, Tian et al. [157] proposed growing AAO on Si substrate with Ti adhesion layer, and then detaching the membrane using this approach. The main advantages of the reverse bias voltage method are its ability to obtain complete AAO membrane and that the same electrolyte used for anodization can also be used for separation, reducing waste and environmental pollution. In 2015, Joo et al. [156] further advanced this method by demonstrating repeated AAO film detachment on the same substrate, leading to its widespread adoption. They used a potential of −15 to −17 V to detach the AAO fabricated in sulfuric acid at 25 V. The schematic diagram of AAO membrane prepared by reverse bias voltage detachment method is shown in Figure 5. Figure 5a represents the aluminum substrate after pre-treatment, which typically undergoes a two-step anodization to form (b) ordered nanoporous AAO structure. During the AAO membrane detachment step shown in Figure 5c, a negative potential of approximately 10–20 V is usually applied to obtain a free-standing AAO membrane. If a through-hole nanostructure is required, the AAO membrane is subjected to phosphoric acid etching to open the pores, as illustrated in Figure 5d. Following the membrane detachment, a pre-texturing concave remains on the aluminum substrate (Figure 5e), which facilitates repetitions of AAO membranes on the same substrate. Obtaining a complete, defect-free membrane from the aluminum substrate is a major advantage of this method, making it even more promising for use in nanomaterial synthesis, filtration, and SERS applications. However, this method still has several drawbacks. First, it is relatively time-consuming, typically requiring over ten minutes in detachment process. Additionally, the gradual increase in the negative voltage requires precise control based on experience. The delamination mechanism of the reverse bias method primarily arises from the applied negative potential, which generates hydrogen gas between the aluminum and the barrier layer, thereby pushing the AAO membrane away. Therefore, the barrier layer is preserved and additional etching is required to achieve a through-hole membrane. These challenges highlight the need for further research to optimize the AAO membrane detachment process.

### 2.4. Pulse Voltage Detachment Method

**Figure 6:**
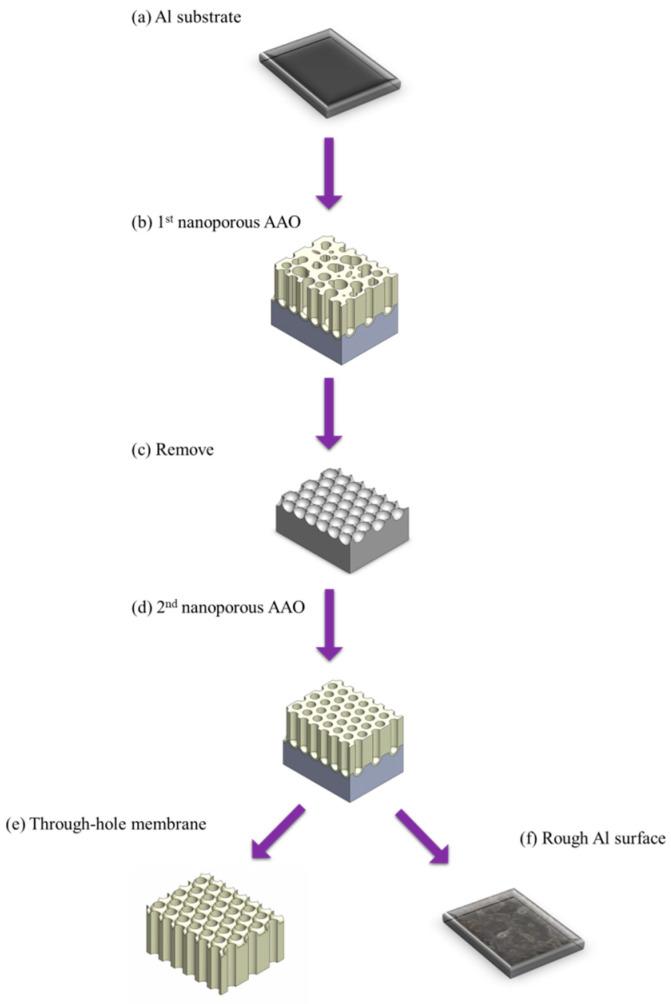
The process flow of AAO membrane fabrication process using pulse voltage detachment method. (**a**) Al substrate, (**b**) 1st anodization with disordered nanoporous AAO, (**c**) remove and leave pre-texturing Al surface, (**d**) 2nd anodization with ordered nanoporous AAO, (**e**) through-hole AAO membrane after detachment process, and (**f**) rough Al surface with impurities.

The pulse voltage method was first proposed by Spooner in 1963 [135]. In 2002, Paterson et al. reported that by applying a voltage of 55 V, which is 15 V higher than anodization voltage in perchloric acid-based solution, the AAO film could be rapidly separated within just a few seconds. In 2006, Xia et al. [169] conducted further research on this method and established that using an ethanol–perchloric acid solution with a voltage 5–15 V higher than the anodization voltage of 40 V enabled rapid AAO detachment. This technique was also known as the single-step simple method, and the mechanism was explained by the dissolution of AAO barrier layer. In the following year, Zhao et al. [168] successfully separated AAO membranes using perchloric acid aqueous solution and determined that a perchloric acid concentration of 60–70% was suitable for the process. In 2015, Sulka et al. [170] further optimized the process parameters and provided a comprehensive explanation of the dissolution mechanism, refining the pulse voltage method. The process flow of AAO prepared by pulse voltage detachment method is shown in Figure 6. The mechanism of pulse voltage detachment method is based on burning effect of barrier layer. When current passes through AAO, the barrier layer presents the lowest resistance, causing a large amount of Joule heating accumulation. The main advantage of this method is its short process time, typically less than 30 s. Moreover, due to the dissolution of barrier layer, through-hole structures are directly formed during the detachment process, without the demand for additional phosphoric acid etching. However, the concentration of high current to barrier layer results in an intense reaction and the generation of mechanical and thermal stress, which compromise the membrane integrity of AAO. Additionally, the rough surface of aluminum substrate makes it unsuitable for repetition on the same substrate. In 2019, Sulka et al. [140] applied pulse voltage detachment to AAO prepared at 25 V in sulfuric acid. Although it presented the first photographs of a fully separated AAO film, the film edges appeared uneven, and repeated AAO membrane detachment experiments on the same substrate were not feasible.

### 2.5. Two-Layer Anodization Method

**Figure 7:**
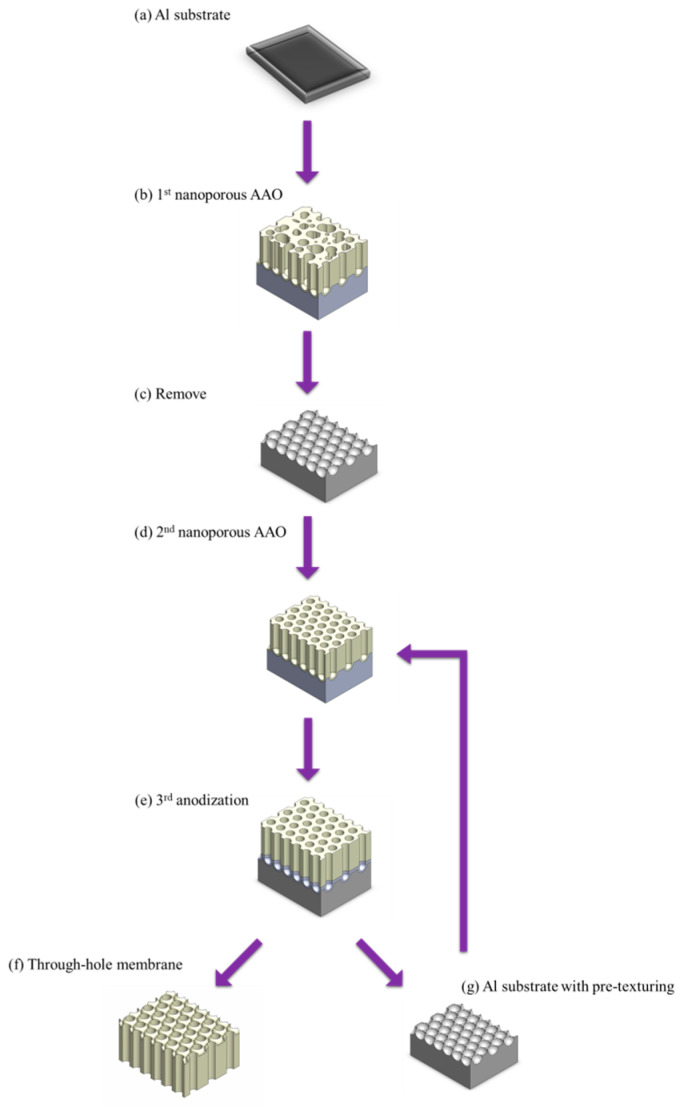
The process flow of two-layer anodization method. (**a**) Al substrate, (**b**) 1st anodization with disordered nanoporous AAO, (**c**) remove and leave pre-texturing Al surface, (**d**) 2nd anodization with ordered nanoporous AAO, (**e**) 3rd anodization to grow sacrificial AAO layer, (**f**) through-hole AAO membrane after detachment process, and (**g**) Al substrate with pre-texturing.

Two-layer anodization method, also known as the further anodization method, achieves AAO membrane detachment by growing a sacrificial layer. In 2015, Masuda et al. [139] proposed to form AAO layer at 40 V in oxalic acid, followed by a thick AAO layer grown in concentrated sulfuric acid (12 M) at 40 V as the sacrificial layer for repetition of AAO membrane. The process flow of two-layer anodization method is shown in Figure 7. Since AAO formed in concentrated sulfuric acid is highly susceptible to phosphoric acid etching, complete AAO membrane detachment is achieved. It offers the advantage of obtaining complete AAO membranes repeatedly, and the etching process results in through-hole nanostructure. In addition, complete membrane is highly important for applications such as nanomaterial synthesis, mask, filtration, and SERS. However, this method requires an additional anodization step and a prolonged etching process, making the overall procedure more complex and time-consuming. Furthermore, the use of concentrated sulfuric acid for anodization raises concerns about its chemical waste and environmental impact. To address this issue, in 2017, Ling et al. [171] proposed an alternative method in which annealing at 500 °C for 2 h is used to densify the original AAO layer prepared at 40 V in oxalic acid. Then, the AAO is further anodized under the same condition to form the sacrificial layer. This approach allows the sacrificial AAO layer to be etched away more rapidly, and avoids the need for concentrated sulfuric acid. However, it still suffers from process complexity, and leads to severe pore-widening effects.

### 2.6. Constant Voltage Detachment Method

The constant voltage membrane detachment method operates at a specific voltage higher than that used during anodization, and the electrolytes used can be categorized into two types of perchloric acid/ethanol and NaCl/ethylene glycol (EG) solutions.

**Figure 8:**
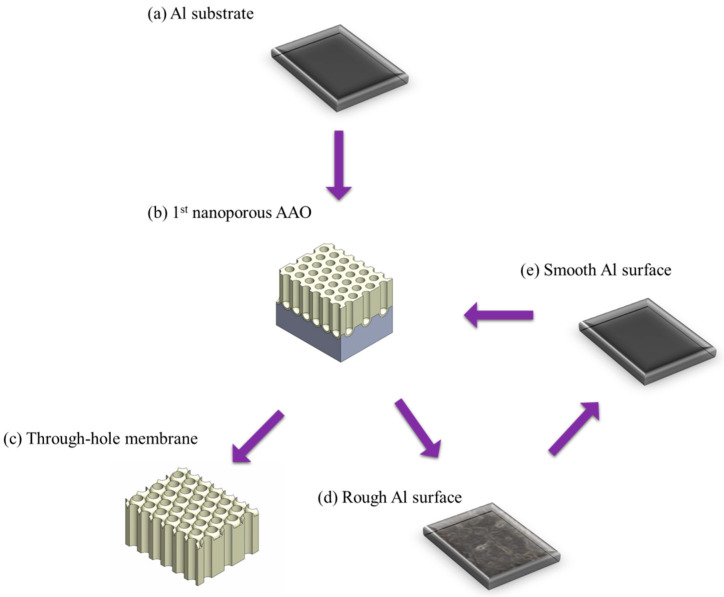
The process flow of constant voltage detachment method by perchloric acid and ethanol solution. (**a**) Al substrate, (**b**) 1st anodization nanoporous AAO, (**c**) through-hole AAO membrane after detachment process, (**d**) rough Al surface with impurities, and (**e**) smooth Al surface after prolonged two-step electropolishing process.

**Figure 9:**
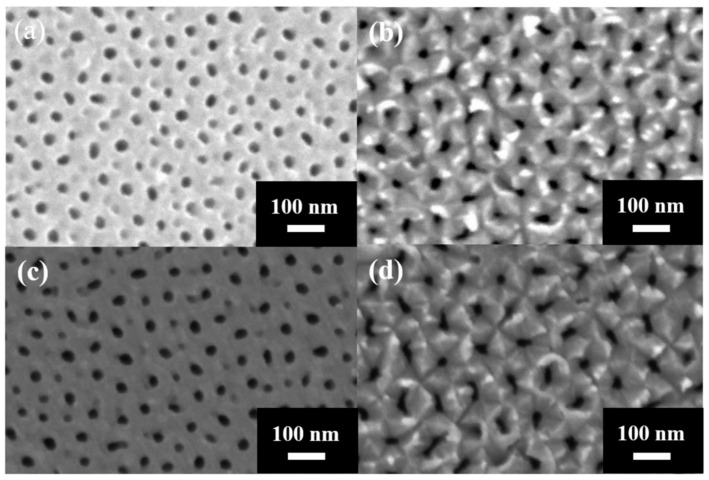
The SEM top and bottom views of AAO membrane multi-detachment in (**a**,**b**) 3rd and (**c**,**d**) 5th repetition by constant voltage detachment method. (From Ku et al., used under CC-BY 4.0 license [175]).

In 2024, Chung et al. [175] proposed a method for AAO membrane detachment by applying 50 V for 20 s at room temperature based on perchloric acid to ethanol solution with 1:1 volume ratio, effectively detaching AAO fabricated at 40 V. The process flow is illustrated in Figure 8. This method is able to preserve the integrity of AAO membrane because it employs lower voltage and longer detachment time to reduce internal stress. To achieve the repetition of membrane detachment process, they also developed a two-step electrochemical polishing process to reduce substrate impurities and surface roughness. The two-step electropolishing is performed by solution of HClO_4_:C_2_H_5_OH = 1:1 for 2 min and HClO_4_:C_2_H_5_OH = 1:4 for 10 min to smooth Al surface. It allows the subsequent repeated detachment process to be carried out five times. The top and bottom views of SEM images from the third and fifth membrane detachment processes are shown in Figure 9.

**Figure 10:**
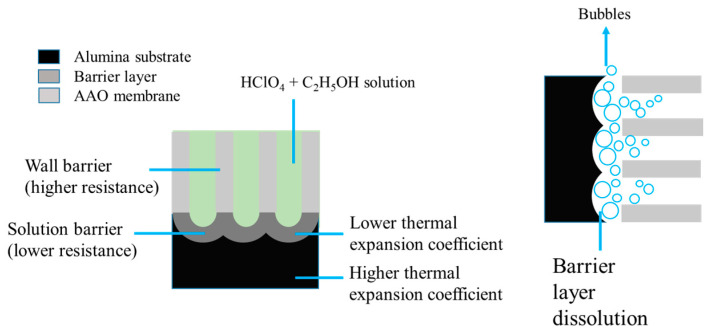
A schematic diagram of membrane detachment mechanism from barrier dissolution, mechanical stress and thermal stress by constant voltage detachment method. (From Ku et al., used under CC-BY 4.0 license [175]).

The detachment mechanism mainly involves barrier layer dissolution due to Joule heating [175], similar to that of the pulse voltage method. The film integrity in this method is primarily influenced by mechanical stress, including gas bubbles generated during the process and the thermal expansion mismatch between materials, as shown in Figure 10 [175]. During the detachment process, bubbles are generated by chemical dissolution. Initially, bubbles form at the lower-resistance barrier and expand toward the barrier/AAO interface, resulting in membrane peeling with uneven vertical-to-membrane gas stress from bubble formation and escape. Another factor generating stress is the difference in thermal expansion coefficients between aluminum and alumina, which are 23.2 × 10^−6^/°C and 7 × 10^−6^/°C, respectively. When the temperature of the barrier layer and Al substrate increases unevenly, additional uneven thermal stress is applied to the Al/AAO interface during peeling. The advantages of this method include a short membrane detachment time, and the formation of through-hole structures without the additional process of phosphoric acid etching. By lowering the applied voltage and extending the processing time, it also reduces mechanical and thermal stress, enabling repeated detachment of complete AAO membranes.

Another constant voltage detachment method uses NaCl/EG solution. In 2022, Kikuchi et al. [173] proposed a constant voltage detachment method using NaCl/EG solution, applying 28–36 V to detach AAO membranes fabricated at 25 V in sulfuric acid. As the detachment voltage increased, the required time decreased from 2.5 s to 0.5 s. In 2023, Kikuchi et al. [174] further proposed detaching AAO membranes prepared in oxalic acid and phosphoric acid by applying a constant voltage 10 V higher than the anodizing voltage. Its benefits include shorter processing time and lower toxicity of the electrolyte. However, due to the low conductivity and corrosiveness of the solution, the AAO barrier layer still remains and requires an additional phosphoric acid etching step for about 15 min.

### 2.7. Summary of AAO Membrane Detachment Methods

The advantages and disadvantages of AAO membrane detachment methods include membrane integrity, process time and complexity, electrolyte toxicity, and substrate reusability. Table 2 summarizes several reported AAO membrane detachment methods based on these criteria, comparing the pros and cons of each. Traditional etching methods typically use heavy metal solutions to dissolve the aluminum substrate and require phosphoric acid etching of the barrier layer, thus offering advantages only in membrane integrity. The gradual voltage decrease method enables intact membrane detachment through an environmentally friendly process, but it suffers from long processing times and lacks literature evidence for membrane reusability on the same substrate. The reverse bias method allows repeated detachment of intact AAO membranes from the same substrate, but it requires a longer detachment time and additional phosphoric acid etching steps. The pulse voltage method is advantageous for its simplicity and fast processing, but the resulting membranes are brittle, and the substrates cannot be reused. Two-layer anodization enables repeated detachment of intact AAO membranes on the same substrate, but it involves a longer process and the use of highly toxic concentrated sulfuric acid. The constant voltage detachment using NaCl/EG solution achieves both membrane integrity and short process time, but it still requires phosphoric acid etching of the barrier layer, and the reusability of the substrate has not yet been reported. The constant voltage method using perchloric acid in ethanol allows simple detachment of intact membranes from the same substrate, but it requires additional electrochemical polishing and involves the use of perchloric acid, which is classified as a strong acid. Although significant improvements have been made across these methods, a process that simultaneously addresses all advantages still requires more comprehensive research.

## 3. Applications of Through-Hole AAO Membranes

AAO membranes have been widely applied in electrical sensors [154,155], nanomaterial synthesis [155,156,157], photomasks [158], filtration [159,160,161,162,163,181], SERS [151,152,153], and TENG [166]. The detached AAO membrane is thin and brittle, so they are not suitable for surface protection applications compared to AAO formed on aluminum substrate. On the other hand, the through-hole structure enables additional applications such as photomasks and filtration. In practical applications, the integrity of the AAO membrane is a critical issue, especially for its use in electrical sensors, nanomaterial synthesis, photomasks, filtration, and SERS, all of which require an intact membrane for effective performance.

### 3.1. Humidity Sensor

The applications of AAO in electrical sensors include humidity sensors [78,79,80], gas sensors [75,76,77], liquid sensors [182], and pressure sensors [183,184,185,186,187]. Current approaches often involve growing AAO directly on aluminum substrates followed by metal deposition to form a capacitor structure, or integrating AAO with other materials to create composite sensors. In 2017, Park et al. [155] fabricated through-hole AAO membranes using the reverse bias method and deposited interdigitated gold electrodes via sputtering for humidity sensing. The capacitive AAO humidity sensor demonstrated a measurement range of RH 30% to 95% with a response of approximately 100%. In 2023, Liu et al. [154] proposed using the stepwise decreasing voltage method to prepare through-hole AAO membranes, followed by spin coating of CNTs, PU, and DMF solution onto AAO to fabricate a composite sensor. It achieved a measurement range of RH 20% to 80%, with a sensitivity of up to 3.51% RH^−1^. Meanwhile, they demonstrated that the AAO sensor exhibited a response–recovery time of less than 2 s when detecting humidity changes in human breath.

The sensing mechanism of AAO humidity sensors can be categorized into three main factors, including external conditions, nanostructures, and anionic effects. External conditions primarily involve changes in humidity and the application of an external magnetic field. In terms of geometrical effects, Chung et al. [76,78] investigated the relationship of anodization voltage and sensor response. They summarized that sensor performance is positive correlated to total pore circumference, or described as specific surface area in AAO. The anionic effect is affected by the concentration of anodization electrolyte. Generally, higher electrolyte concentration leads to larger anionic effect in AAO pore wall, which enhances the sensor performance. However, the aforementioned mechanism has no clear relevance to humidity sensors based on through-hole AAO membranes. The nanostructure of the through-hole AAO membrane does not provide better sensing performance compared to AAO on aluminum substrates. Compared with other materials, the advantages of the AAO humidity sensor are the simple fabrication process and stability of oxide [188]. However, fabricating humidity sensors based on through-hole AAO structures increases the complexity of the manufacturing process, which has resulted in fewer research efforts and limited breakthroughs in the development of AAO-based humidity sensors.

### 3.2. Nanomaterial Synthesis

AAO is an ordered nanomaterial template and commonly used for the synthesis of nanomaterials such as metals, oxides, polymers, and carbon-based composites [189,190,191,192,193]. The synthesized materials can be applied more broadly, for instance, the carbon-based materials derived from AAO can make significant contributions in energy applications [189,190]. The synthesis process includes physical vapor deposition, chemical vapor deposition, atomic layer deposition (ALD), sol–gel, hydrothermal, electroless and electrodeposition techniques. By combining AAO template for synthesis, it offers advantages such as simplicity, low cost, and ability to synthesize a wide variety of nanostructures, including nanowires (NWs), nanotubes (NTs), nanodots (NDs), and nanofilms [67,68,69,140,155,157,189,190,191,192,193,194]. Except traditional AAO on bulk Al, through-hole AAO membranes can also be used for nanomaterial synthesis.

**Figure 11:**
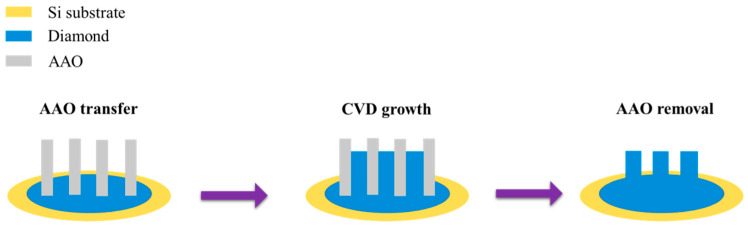
The process flow of diamond nanomaterial synthesis by AAO membrane and CVD process.

In 2017, Park et al. [155] proposed using the reverse bias method to detach AAO membranes, followed by phosphoric acid etching for 5–55 min to produce through-hole AAO membranes with varying pore diameters. They utilized an electrochemical polymerization method to synthesize light-emitting poly(3-methylthiophene) and conducting polypyrrole nanowires with diameters ranging from 25 to 50 nm. Additionally, they demonstrated the synthesis of polypyrrole nanowires with diameters of 140–250 nm using commercially available AAO membranes. In the same year, Vega et al. [192] proposed separating AAO membranes using an HCl and CuCl_2_ solution, followed by ALD synthesis of various metal oxides (Al_2_O_3_, SiO_2_, TiO_2_, Fe_2_O_3_, ZnO), and investigated their diffusion rates. The results demonstrated precise control and improved membrane performance for specific applications. In 2019, Sulka et al. [140] proposed another work of nanomaterial synthesis by pulse voltage detachment method and electrodeposition. After membrane detachment, a thin Au layer was first deposited onto the AAO membrane by sputtering, followed by electrodeposition of gold nanowires at −4 mAcm^−2^. Finally, the AAO was etched using 1.0 M NaOH to obtain the synthesized Au nanowires. Also in 2019, Palmero et al. [193] also demonstrated the use of AAO membranes combined with ALD for nanomaterial synthesis. They successfully synthesized and controlled the diameter of Ni and Fe-Co nanowires, achieving the desired magnetic properties. In 2023, Buijnsters et al. [67] reported the growth of diamond nanopillar structures using AAO membranes. First, a polycrystalline diamond film was coated onto a silicon substrate. Then, a commercial AAO membrane was placed on top of the diamond film, followed by CVD method for diamond growth at 725 °C from 0.5 to 3 h. Finally, the AAO membrane was removed by phosphoric acid etching at 200 °C for 2 h, resulting in a nanopillar diamond structure. By using different templates and parameters, diamond nanopillars with diameters of approximately 300 nm and 70 nm were successfully fabricated. The experimental procedure is redrawn in Figure 11.

Although current applications of AAO through-hole membranes have demonstrated the successful synthesis of various nanostructured materials, AAO formed on aluminum substrates also performs well for similar applications. As a result, the necessity of fabricating free-standing AAO membranes remains unclear. However, due to the diverse requirements for different nanomaterials and nanostructures, AAO membranes still hold significant potential for applications in nanomaterial synthesis.

### 3.3. Mask

**Figure 12:**
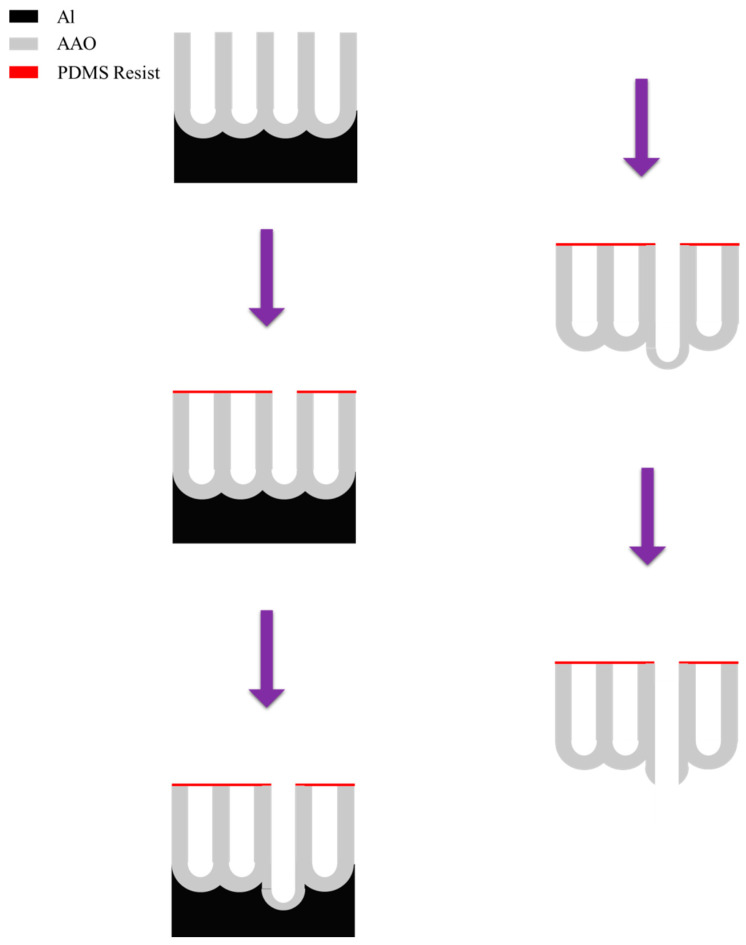
The process flow for fabrication of AAO membrane masks by etching and two-layer anodization method.

Another appropriate application of AAO membranes is mask, first proposed by Masuda et al. [158] in 2018. This technique employs etching method and two-layer anodization process for AAO mask fabrication. The process flow is redrawn in Figure 12. To fabricate an ordered pore array, the aluminum substrate was first pre-imprinted with a Ni mold to create regularly spaced indentations with a 500 nm pitch. Anodization was then carried out at 200 V in 0.1 M phosphoric acid to grow a well-ordered pore structure. Subsequently, a resist mask was formed on the alumina surface using PDMS combined with electron beam lithography and dip-coating. A sacrificial layer was then created by constant-voltage anodization at 200 V in 17.6 M sulfuric acid at 0 °C. Finally, the aluminum substrate and the sacrificial AAO layer prepared in sulfuric acid were etched using a saturated solution of iodine in methanol and 10 wt% phosphoric acid, respectively, yielding an AAO membrane photomask with pores at designated positions.

### 3.4. Filtration

**Figure 13:**
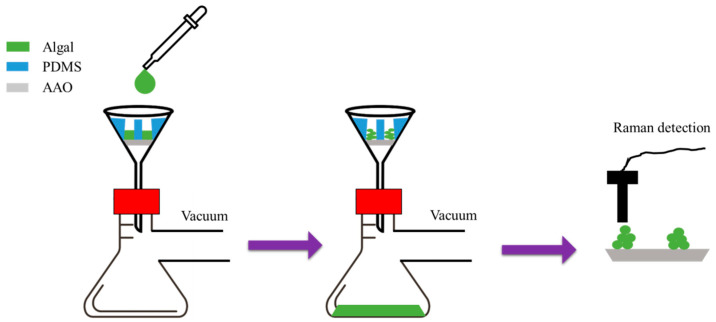
The process flow of AAO membrane application in algal paste filtration and detection.

The application of AAO membranes in filtration can be broadly categorized into flat or tubular AAO membranes. The first approach involves detaching the AAO membrane and using it for filtering specific substances. This method typically applies flat and small-area AAO membranes. For instance, Juang et al. [159] proposed using PDMS microwell combined with commercial AAO membrane with pore diameter of 25 nm for algal paste filtration, as illustrated in the experimental procedure shown in Figure 13. By using a Buchner flask and low vacuum method, uniform algal cells can be rapidly collected. Moreover, the excellent biocompatibility and low background noise of the AAO membrane are advantageous for Raman analysis.

**Figure 14:**
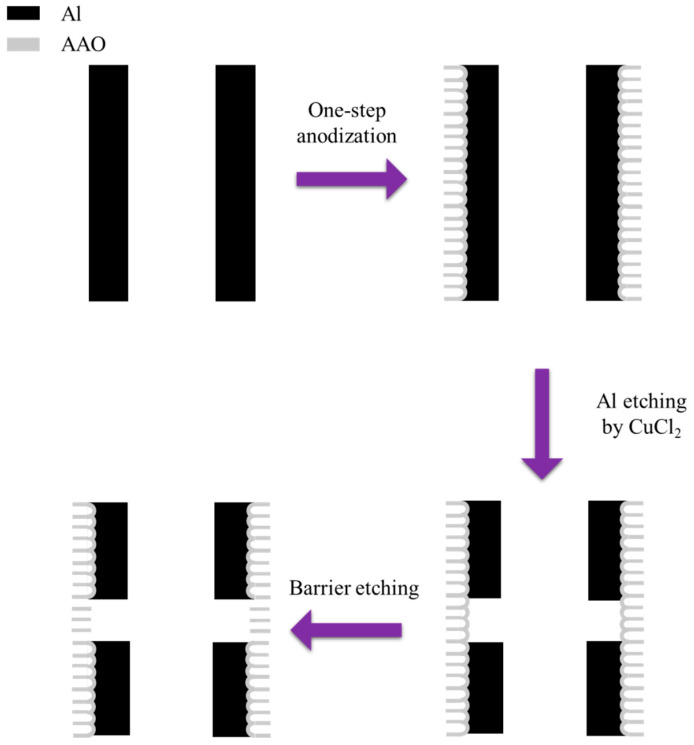
The process flow for tubular AAO membranes fabrication by etching.

The other approach is based on growing AAO on cylindrical aluminum products, followed by etching to remove the aluminum, resulting in tubular AAO membranes [161,162,163]. In 2021, Weng et al. proposed fabricating tubular AAO membranes by performing one-step anodization on a 6063 aluminum alloy tube in 0.3 M oxalic acid at 6–7 °C for 8 h [163], as shown in Figure 14. A silicone tube was then used to protect the inner layers at both ends of the aluminum tube during etching with CuCl_2_ solution to remove the aluminum. Subsequently, the barrier layer was etched using a 6 wt% phosphoric acid solution at 30 °C to obtain an AAO membrane connected to the aluminum tube. The average pore size of AAO membrane could be increased from original 35 nm to a maximum of 109 nm through phosphoric acid etching. This technology shows great potential for applications in the bionic, biomedical, and chemical fields.

### 3.5. Raman and Surface-Enhanced Raman Scattering (SERS) Applications

Raman spectrum and SERS are powerful analytical techniques that has been widely applied in the detection of dyes, drugs, antibiotics, and preservatives [195]. SERS detection methods can generally be categorized into two types; colloidal solution [196,197,198] and solid substrate [83,84,199,200]. Although metallic nanoparticles in solution offer high sensitivity, their uniformity and reproducibility are limited. On the other hand, solid substrates provide better stability but often compromise SERS performance. To advance SERS using solid substrates, developing strategies to enhance signal through special nanostructures has become an important research direction. In AAO-based substrates, the three-dimensional structures formed at the bottom during membrane detachment present a promising solution, and several studies have already been conducted in this area.

Research on free-standing AAO membranes as solid substrates can be divided into two categories, as listed in Table 3 for comparison. The first involves applications of filtration and Raman detection by AAO membrane [159,201]. In 2025, Shin et al. [201] proposed using through-hole AAO membranes for filtration and Raman signal detection of nanoplastics. The fabrication process still employed the traditional two-step anodization followed by CuCl_2_-based solution for Al substrate etching and phosphoric acid etching for pore opening to obtain the through-hole membrane structure. This method successfully identified six types of nanoplastics, including polyethylene (PE), polypropylene (PP), polyethylene terephthalate (PET), poly(methyl methacrylate) (PMMA), polystyrene (PS), and polylactic acid (PLA), on AAO membranes with pore diameters ranging from 20 to 160 nm, with clearly distinguishable peaks to verify different nanoplastics. This research supports future applications of AAO membranes in filtration and Raman-based analysis for water quality and environmental monitoring.

**Figure 15:**
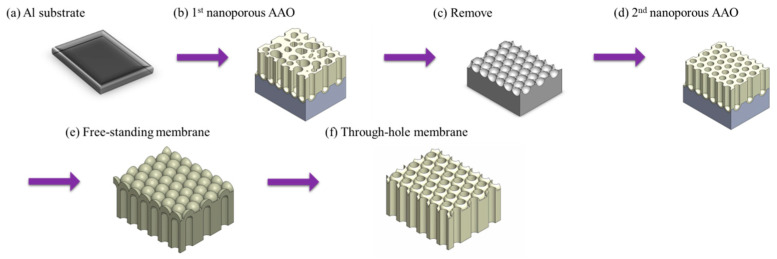
The process flow of through-hole AAO membrane preparation.

The other approach utilizes the nanostructures on the bottom of AAO membrane to create additional hotspots, thereby further enhancing the SERS signal for different substance detection [151,152,153,202]. In 2018, Buyukserin et al. [152] proposed using the conventional two-step anodization method in oxalic acid to fabricate 30 μm thick AAO, followed by etching the aluminum substrate and the barrier layer with CuCl_2_ and a mixed solution of phosphoric and sulfuric acids, respectively, to obtain a through-hole AAO membrane. The fabrication process is described in Figure 15. A thin gold layer of 10–30 nm was then deposited on the bottom of AAO membrane via physical vapor deposition, and the substrate was used for detecting methylene blue (MB) and Congo red (CR) to 10^−7^ M. This method successfully demonstrated that the through-hole structure on bottom of AAO enhances the SERS signal, with the optimal performance observed at a gold thickness of 20 nm. Another example is the study by Kim et al. [202], who proposed using AAO membranes to synthesize nanoscale dendritic silver and gold nanoparticles for SERS detection of 4-methylbenzenethiol (4-MBT). The fabrication process involved a two-step anodization combined with current limitation to reduce the anodization voltage, thereby thinning the barrier layer and lowering resistance. Subsequently, alternating current electrodeposition of silver was performed at 7 V, followed by etching to remove the aluminum substrate and any remaining barrier layer. Finally, gold was sputter-coated onto the silver nanostructures at the bottom of the AAO membrane, forming a unique structure of nanobranch Ag and Au nanoparticles.

### 3.6. Tribo-Electrical Nano-Generators (TENG)

**Figure 16:**
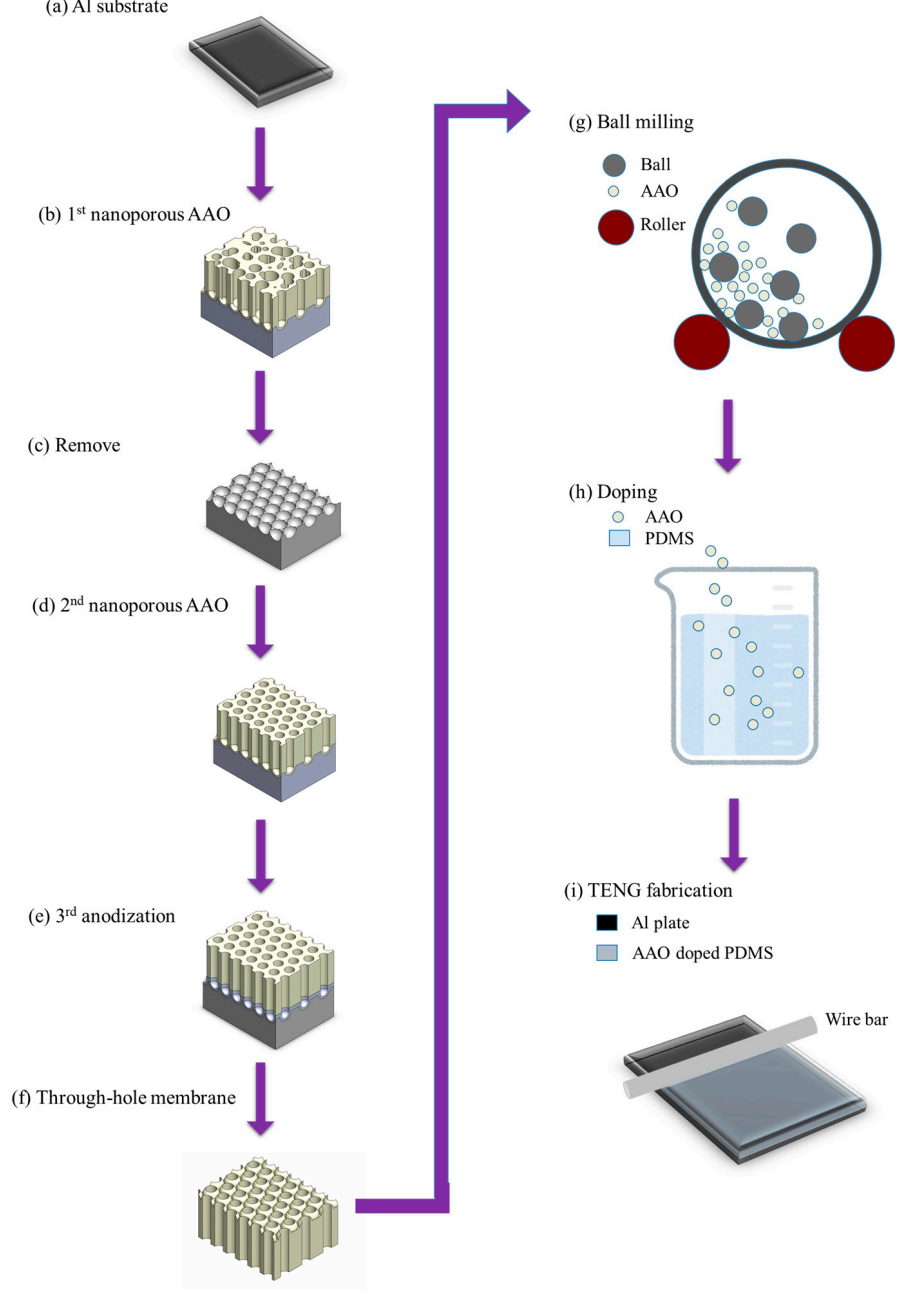
The process flow of AAO nanoparticle-doped TENG fabrication.

TENG is an emerging green energy technology that converts mechanical energy, wind, and water from the environment into electrical energy [203,204,205,206]. Current research primarily focuses on improving power generation efficiency, developing self-powered sensors, wearable devices, and human–machine interfaces [207,208,209,210]. In terms of power output, many research teams have explored surface structure modification and doping with dielectric or conductive materials [166,211,212,213]. In 2024, Choi et al. [166] proposed enhancing power generation performance by detaching and grinding AAO membranes into nanoparticles, then doping into PDMS. The experimental procedure is illustrated in Figure 16. They employed a conventional two-step anodization process to grow AAO in 0.3 M oxalic acid, then used a double-anodization method to grow a sacrificial layer in 12 M sulfuric acid at 0 °C under 50 V for 20 min, followed by etching to obtain the AAO membrane. The free-standing AAO membrane was then ground for 12 h using a ball milling method to produce AAO nanoparticles, which were subsequently incorporated into PDMS to fabricate AAO-doped TENG. The study showed that doping with 7 wt% AAO nanoparticles yielded the best TENG output, reaching 130 V and 3 μA, which is approximately three times higher than that of undoped pure PDMS. This research did not focus on preserving the integrity of AAO membrane after detachment, but instead ground it into particles, demonstrating a new application direction.

### 3.7. Summary of Applications of Through-Hole AAO Membranes

Various methods for AAO membrane detachment and their applications have been extensively developed, including humidity sensors, nanomaterial synthesis, mask, filtration, SERS detection and TENG, as discussed in detail in Section 3.1, Section 3.2, Section 3.3, Section 3.4, Section 3.5 and Section 3.6. However, the extent of research development using AAO membranes varies significantly, mainly due to the suitability of the membranes for specific applications. For example, in the cases of humidity sensors and nanomaterial synthesis, the use of AAO membranes does not offer a significant advantage over AAO formed on bulk aluminum, thereby limiting their research value. The application of AAO membranes as mask represents a novel direction, but due to the complexity of the fabrication process and the high cost of equipment, related work remains limited. On the other hand, AAO membranes show applicability in filtration and as substrates for Raman or SERS detection. In filtration applications, the AAO structures formed on aluminum metal cannot achieve through-hole characteristics, highlighting the necessity of membrane detachment processes. Moreover, the tunable pore structure of AAO offers the potential to filter various substances. However, current studies remain in the research stage, and practical applications such as water filtration are still underdeveloped, representing a promising area for future advancement. The use of AAO membrane nanostructures in SERS is also a direction that should not be overlooked. The nanostructures formed on the bottom side of AAO membrane can provide more hotspots, significantly enhancing the SERS signal compared to AAO on aluminum surfaces. Nevertheless, related studies still rely on relatively time-consuming membrane detachment processes, and the effects of different AAO fabrication parameters on membrane structures have not been explored, indicating that further investigation is valuable. Finally, the application of AAO membranes in TENG is among the few areas where the integrity of the free-standing AAO membrane is not critical, opening a new direction. However, various materials can be doped into TENGs, including carbon-based materials, TiO_2_, and Al_2_O_3_. The necessity of specifically separating and grinding AAO membranes for doping, as well as the performance comparison among different doped materials, still requires further study.

Except for the applications mentioned above, the tunable vertically aligned porous structure of AAO films holds promise for future applications in water purification, substance separation, and various sensor substrates. Although several studies have reported the use of AAO in filtration applications, to our best knowledge, the research for water purification has not been proposed. This limitation arises because AAO cannot efficiently remove contaminants and impurities in water. Similarly, substance separation remains an area requiring further development, as different separation conditions demand precise control over pore morphology and surface characteristics. Effective approaches may therefore include modulating pore geometry and integrating AAO with functional materials. Another future direction lies in the optical applications of AAO membranes. After removing the aluminum substrate, the resulting optical transmittance enables a variety of advanced detection techniques, such as transmittance spectroscopy, or Fourier-transform infrared spectroscopy (FTIR), which could be potentially be combined with AAO membranes for specific substance identification. Furthermore, by grinding AAO membranes and exploiting their inherent photoluminescence (PL) properties, it is possible to develop invisible inks, offering an additional novel application for AAO membranes.

## 4. Conclusions

In this review, we thoroughly introduce the differences between AAO on aluminum substrates and free-standing AAO membranes, as well as several detachment methods and applications of AAO membranes. Recent research on AAO membranes has focused on developing fast, simple, and repeatable methods for obtaining complete membranes, along with innovative and effective applications. The detachment methods for AAO membranes can be categorized into etching-based and voltage-based approaches. Etching methods can be further divided into aluminum substrate etching and the two-layer anodization technique. Traditionally, membrane detachment has been achieved using HgCl_2_ or CuCl_2_ solutions, which can produce large-area and complete membranes. However, this method results in significant substrate waste, time-consuming, and environmental concerns. The two-layer anodization method introduces a sacrificial layer to repeatedly obtain complete membranes from the same substrate. Nonetheless, it requires a total of three anodization steps, making the process complex and time-consuming. Voltage-based detachment methods include voltage reduction method, reverse bias detachment method, pulse voltage detachment, and constant voltage techniques. However, stress generated during the process poses challenges to maintaining membrane integrity. Among them, the reverse bias detachment method enables repetition of complete membranes from the same substrate, but it is relatively time-consuming and requires an additional barrier layer etching step. The pulsed voltage method offers a simple and rapid process, but it struggles to retain membrane integrity. The constant voltage method, depending on the electrolyte used, often requires additional polishing or phosphoric acid etching steps to achieve through-hole structures. Although the aforementioned methods have achieved certain improvements, a method that combines all the advantages has not been developed. Therefore, further research in this area holds significant value. In the applications of AAO membranes, whether they can replace the performance of AAO on aluminum substrates is a crucial indicator. Although some research teams have proposed applications in nanomaterial synthesis and humidity sensors, the performance has not surpassed that of AAO on aluminum substrates. On the other hand, in applications such as filtration and SERS substrates, the nanoporous structure of AAO membranes offers unique advantages, prompting more teams to conduct effective research. Most applications require the preservation of complete AAO membranes. In applications of humidity sensors, nanomaterial synthesis, photomasks, filtration, and SERS, the integrity of AAO membrane addresses a critical issue. On the other hand, grinding AAO into nanoparticles as a dopant in TENG devices presents a novel direction. In addition to the applications mentioned above, the potential of AAO combined with various fabrication processes for synthesizing functional nanomaterials, such as semiconductor materials, carbon-coated composites, and magnetic materials, should not be underestimated. The tunable vertically aligned porous structure of AAO films holds promise for future directions in water purification, substance separation, and various optical applications. Although there are still challenges for AAO membrane research, the widely tunable nanostructure and its high-performance applications will play a crucial role in environmental safety, bio-applications, energy technologies, and food safety.

## Figures and Tables

**Table 1:** A brief comparison of traditional etching process for AAO membrane detachment.

Solution	Parameters	Ref.
HgCl_2_	saturated HgCl_2_ solution	[113,121,122,123,124,125,126]
	saturated HgCl_2_ solution for 10 h	[127]
	2% HgCl_2_ solution	[128]
CuCl_2_-based solution	saturated CuCl_2_ solution	[129]
	100 mL HCl (38%) +100 mL H_2_O +3.4 g CuCl_2_·2H_2_O at 15 °C for 3 h	[130]
	100 mL HCl (38%) +100 mL H_2_O +3.4 g CuCl_2_·H_2_O at room temperature for 10 min	[131]
	solution of HC1 (32%) and CuCl_2_ (0.05%)	[132]
	solution made up of one part by volume of 0.1 mol/L CuCl_2_ solution and four parts by volume of 10 wt% HCl	[133]
	CuCl_2_ & HCl solution	[177]
Iodine methanol solution	saturated iodine methanol solution at 50 °C	[178]
	saturated iodine methanol solution	[179]

**Table 2:** Comparison of AAO membrane detachment methods and results.

Ref.	Method	Al Purity	Anodization Step/Solution	AAO Fabrication Time (h)	Detachment Time	Photographs of Complete Membrane	Repetitions of AAO Membrane
[176] (Commercial membrane)	Chemical etching method	99.999%	NA	NA	NA	Complete	NA
[136]	Voltage reduction method	99.95%	1-step/Phosphoric acid	1.25	NA	Complete	NA
[156]	Reverse bias voltage method	99.999%	2-step/Sulfuric acid	25	20 min	Complete	6 times
[157]	Reverse bias voltage method	99.999%	2-step/Sulfuric acid	44	13 min	Local *	NA
[167]	Reverse bias voltage method	99.999%	2-step/Oxalic acid	8–32	30–90 s	Local *	5 times
[168]	Pulse voltage method	99.999%	2-step/Oxalic acid	28	3 s (1 cycle)	Local *	NA
[169]	Pulse voltage method	99.99%	2-step/Oxalic acid	5	3 s (1 cycle)	Local *	NA
[170]	Pulse voltage method	99.999%	2-step/Oxalic acid	5	3–60 s (1–10 cycle)	Local *	NA
[140]	Pulse voltage method	99.999%	2-step/Sulfuric acid	12–20	3–60 s (1–10 cycle)	Partial	NA
[139]	Two-layer anodization method	99.999%	3-step/Sulfuric acid	13.5	15 min	Complete	10 times
[171]	Two-layer anodization method	99.999%	3-step/Oxalic acid	27	75 min	Complete	4 times
[173]	Constant voltage detachment method	99.999%	NA/Sulfuric acid	NA (thickness of 20~80 μm is controlled)	<10 s	Complete	NA
[174]	Constant voltage detachment method	99.999%	NA/Sulfuric acid and Oxalic acid and Phosphoric acid	NA (thickness of 20 μm is controlled)	0.5~20 s	Complete	NA
[175]	Constant voltage detachment method	Al 1050 alloy (~99.5%)	1-step/Oxalic acid	3	20 s	Complete	5 times

* SEM only.

**Table 3:** The comparison of solid substrates prepared by AAO free-standing membrane for Raman and SERS applications.

Ref.	Substrate Material	Fabrication/Detachment Method	Metal Coating	Detection Substance	LOD
[201]	AAO through-hole membrane	Two-step anodization/etching	NA	PE, PP, PET, PMMA, PS, and PLA nanoparticles	NA
[151]	AAO free-standing membrane (with barrier layer)	Two-step anodization/etching	Au	AFB_1_ ZON DON	1.8 ng/mL 47.7 ng/mL 24.8 ng/mL
[152]	AAO through-hole membrane	Two-step anodization/etching	Au	MB and CR	10^−7^ M
[153]	AAO through-hole membrane	Two-step anodization/pulse voltage detachment	Ag	AFB_1_	0.01 ng/mL
[202]	AAO free-standing membrane	Two-step anodization/etching	Ag and Au	4-MBT	1 mM

## Data Availability

Data are presented in the coauthors’ research results, and the schematic drawing is available upon request.
